# Adherent Natural Killer Cells De Novo Express IL-2Rα and Sustain Long-Lasting, Potent Anti-Tumor Activity in Picomolar Concentrations of IL-2

**DOI:** 10.33696/cancerimmunol.7.110

**Published:** 2025

**Authors:** Nikola L. Vujanovic, Lazar Vujanovic, Theresa L. Whiteside

**Affiliations:** 1 UPMC Hillman Cancer Center, Pittsburgh, PA, USA; 2 Department of Pathology, University of Pittsburgh, Pittsburgh, PA, USA; 3 Department of Otolaryngology, University of Pittsburgh, Pittsburgh, PA, USA; 4 Department of Immunology, University of Pittsburgh, Pittsburgh, PA, USA

**Keywords:** IL-2, IL-2 receptors, Activated NK cells, Adherent NK cells, Proliferation, Cytotoxicity

## Abstract

Natural killer (NK) cells are innate lymphoid cells (ILCs) that play key roles in immunosurveillance and immunoregulation. They constitute a heterogeneous population comprising three principal subpopulations: NK1 (cytotoxic), NK2 (regulatory), and NK3 (adaptive). In response to interleukin-2 (IL-2) stimulation, NK3 cells differentiate into adherent NK (A-NK) cells, which exhibit potent anti-tumor activity. Human A-NK cells are generated by priming and adherence-based selection of peripheral blood NK3 cells in nanomolar (nM) IL-2 concentrations, followed by prolonged restimulation and culture in the same IL-2 conditions. However, these A-NK cells are terminally differentiated, unresponsive to IL-2, prone to apoptosis, and are ineffective in cancer therapy. Here, we report previously unrecognized physiological properties of A-NK cells and describe a novel strategy for their *in vitro* generation. Specifically, we demonstrate that A-NK cells primed with nM IL-2 concentrations *de novo* express the high-affinity IL-2 receptor (IL-2Rαβγ). Upon subsequent transfer to picomolar (pM) IL-2 concentrations, these cells undergo sustained vigorous proliferation and retain robust anti-tumor activity in long-term cultures. These findings underscore the functional plasticity of NK3 cells, demonstrating that nM IL-2 priming can reprogram them to function efficiently in pM IL-2 as highly effective anti-tumor effectors. This cytokine-mediated reprogramming of NK3 cells provides a physiologically relevant strategy for generating fully functional therapeutic NK cells with reduced IL-2 dependency. This approach offers a promising venue for advancing NK cell-based cancer immunotherapies.

## Introduction

Natural killer (NK) cells are innate lymphoid cells (ILCs) that inherently possess the capacity to kill pathogen-infected and cancerous cells and regulate immune functions [1–6]. Consequently, NK cells serve as key immunosurveillance effectors, playing a critical role in preventing tumor initiation and metastasis. Accordingly, they also represent promising therapeutic agents in cancer treatment [7]. Human peripheral blood contains three main subpopulations of NK cells: NK1 (cytotoxic), NK2 (regulatory), and NK3 (adaptive) cells [1–15]. This classification is based on differential expressions of various adhesion molecules, regulatory receptors, gene expression profiles and functional characteristics, as summarized in Table 1.

NK1 cells constitute approximately 60% of the peripheral blood NK-cell population. They mediate robust NK cytotoxicity and antibody-dependent cell-mediated cytotoxicity (ADCC). Moreover, they express the intermediate-affinity IL-2Rβγ complex and respond to nM concentrations of IL-2 by rapidly acquiring potent, broad-spectrum tumoricidal activity, previously referred to as lymphokine-activated killer (LAK) activity. However, NK1 cells exhibit limited proliferation and cytokine secretion capacities [1,4–6,8,13,14].

NK2 cells account for approximately 10% of the total peripheral blood NK cell population. Upon stimulation, NK2 cells secrete large amounts of immunoregulatory cytokines and consequently modulate immune functions [1,4–6,8,13,14]. Specifically, due to their expression of the high-affinity IL-2Rαβγ complex, NK2 cells respond to pM concentrations of IL-2 with cytokine secretion and proliferation [1–3]. However, owing to their low or absent expression of perforin, granzymes, and CD16, NK2 cells are poor mediators of NK cytotoxicity and ADCC.

NK3 (adaptive) cells comprise approximately 30% of the peripheral blood NK-cell population. They mediate robust NK activity but exhibit poor ADCC [1,4,6,8–11,13,14]. They also preferentially mount robust recall responses upon viral infection, contact hypersensitivity reaction, and stimulation by pro-inflammatory cytokines or triggering NK-activating receptors [1,9]. Moreover, upon IL-2 stimulation, NK3 cells differentiate into adherent NK (A-NK) cells [10–12]. This process occurs through rapid priming and transient adherence of NK3 cells, followed by vigorous proliferation and robust expansion, secretion of large amounts of immunoregulatory cytokines, enhancement of NK activity, and development of LAK cytotoxicity. Compared to similarly stimulated non-adherent NK (NA-NK) cells, composed predominantly of NK1 and NK2 subsets, A-NK cells demonstrate superior infiltration and tumoricidal activity in solid tumor spheroids *in vitro*. They also exhibit enhanced selective migration into and infiltration of solid tumors *in vivo*, as well as superior antitumor activity in both syngeneic and xenogeneic (human) tumor murine models [16–20]. These prominent antitumor activities of A-NK cells observed in preclinical studies suggest that these cells have an excellent therapeutic potential for the treatment of solid tumors. However, the translation of this promising therapy to clinical practice has failed thus far. The primary barrier to translation has been the inability to manufacture therapeutically sufficient quantities of A-NK cells retaining high levels of cytolytic activity. Furthermore, in the limited number of cancer patients who have received A-NK cell therapy, no detectable tumor response has been observed (our unpublished data).

The objective of this study was to identify the causes underlining the failure to expand therapeutically effective A-NK cells using previously established protocols [10,21], and to develop modified culture protocols that could enhance the translational potential of adoptive A-NK cell therapy for clinical application. To address the observed inability to generate A-NK cells exhibiting robust cytolytic activity, we systematically re-evaluated and optimized the existing culture protocols. Here, we report that A-NK cells cultured for an extended time in the presence of 22 nM IL-2 concentrations, following previously established protocols, undergo terminal differentiation, become unresponsive to IL-2, and are susceptible to pro-apoptotic signals. These findings provide a mechanistic explanation for the limited expansion and reduced therapeutic efficacy of A-NK cells generated using standard protocols. Importantly, we show that A-NK cells primed with nanomolar (nM) concentrations of IL-2 *de novo* express the high-affinity IL-2 receptor (IL-2Rαβγ). Subsequently, reducing IL-2 to pM concentrations during culture is essential to prevent their terminal differentiation, support sustained proliferation, and preserve robust antitumor cytotoxicity. The translational modulation of IL-2 receptor expression during A-NK cell priming, as described in this manuscript, provides a framework for the consistent, large-scale generation of functionally potent and therapeutically promising A-NK cells.

## Materials and Methods

### Reagents and antibodies

Reagents and antibodies used in this study are listed in Supplemental Table 1.

### Cell lines

Human K562 and Daudi cell lines were obtained from ATCC (Manassas, VA). They were cultured in RPMI-1640 medium, supplemented with 10% (v/v) fetal bovine serum (FBS; RPMI-10% FBS), L-glutamine, antibiotics, and HEPES buffer (Thermo-Fisher). All cultures were tested for *Mycoplasma* using the Lonza Mycoplasma Detection Kit (Thermo-Fisher) and were found to be negative.

### Isolation of peripheral blood mononuclear leukocytes and NK cells

Peripheral blood mononuclear leukocytes (PBMLs) were isolated from healthy donor buffy coats (obtained from the Central Blood Bank, Pittsburgh, PA) using Histopaque^®^−1077 density gradients (Sigma-Aldrich). NK cells were isolated from PBMLs using human NK Cell Isolation Kit (Miltenyi Biotec, San Diego, CA), following the manufacturer’s protocol. The purity of isolated CD56^+^CD3^−^CD14^−^CD19^−^CD11c^−^ NK cells was ≥92% as previously reported [22].

### Priming and adherence selection of A-NK and non-adherent NK (NA-NK) cells

Isolated NK cells (10^6^/mL) were resuspended in complete cell culture medium (CM), composed of RPMI-1640 medium supplemented with 2 mM L-glutamine, 50 U/mL penicillin, 50 μg/mL streptomycin, 250 μg/ml Fungizone, 25 mM HEPES buffer (Thermo-Fisher) and 10% human AB serum (Millipore Sigma, Rockville, MD). NK cell suspensions were seeded in tissue culture plates or T25 flasks (CORNING, Corning, NY), supplemented with 22 nM (6,000 IU) of IL-2, and incubated at 37°C for 5 h. Following incubation, A-NK cells were separated from NA-NK cells by first collecting medium containing floating NA-NK cells and then removing residual NA-NK cells through five washes with pre-wormed (37°C) RPMI-1640 medium supplemented with 1% AB serum.

### Enumeration of A-NK cells following selection by adherence

A-NK cells were counted *in situ* in seven randomly selected microscopic fields using an Olympus inverted microscope equipped with 200x magnification lens and ocular grid. The total number of A-NK cells per culture plate was calculated using the following formula:

$$\text{[(A-NK cell number in a microscopic field/0.201 × 200)}\times \text{area (}{\text{cm}}^{2}\text{) of plate surface]},$$

where “0.201” represents the area of ocular grid in cm^2^ and “200” is the magnification factor. Based on the A-NK cell counts, the percentage of A-NK cells within the whole NK-cell population was calculated. In some experiments, A-NK cells were collected as cell suspensions after their incubation in Ca- and Mg-free phosphate-buffered saline (PBS, Thermo-Fisher) at 4°C for 30 min.

### Cultures of A-NK cells and NA-NK cells

Following their priming and selection by adherence, A-NK cells and NA-NK cells were cultured using several different conditions. (i) A-NK cells and NA-NK cells were continuously cultured for 14 to 18 days in CM containing 22 nM IL-2. (ii) A-NK cells were cultured with 22 nM IL-2 for 10 or 14 days, washed and then re-cultured in CM supplemented with titrated IL-2 concentrations (0.022–222 nM) for 4 days. (iii) A-NK cells were cultured with titrated IL-2 concentrations (0.022–22 nM) for 4 to 5 days. (iv) A-NK cells were cultured with 0.022 nM IL-2 for 0, 2, 4, 5, 6, 8 or 10 days, and then re-cultured with 22 nM IL-2 till day 13 of culture.

### Flow cytometry

NK cells (0.2 × 10^6^/0.1 mL) were suspended in ice-cold FACS buffer (PBS containing 0.1% sodium azide and 1% FBS). Cells were incubated on ice for 30 min in the dark with fluorochrome-conjugated mAbs or matching isotype controls per manufacturers’protocols. Subsequently, cells were washed twice with FACS buffer, fixed in 1% (w/v) paraformaldehyde/PBS solution, and analyzed by flow cytometry, as previously described [10]. Flow cytometry analyses were performed on a BD Accuri^™^ C6 cytometer (Beckman Coulter, Brea, CA). Data were computed using FlowJo v10 software (FlowJo, LLC; Ashland, OR).

### Proliferation of A-NK cells

Standard ^3^H-thymidine incorporation assays were used to measure NK-cell proliferation [23]. Typically, the assays were performed in flat-bottom 96-well plates by culturing 5,000 NK cells/200 mL CM, without or with IL-2 (0.022–222 nM) in 4 to 6 replicates. For the last 4 h of these cultures, NK cells were incubated with 2 μCi of [^3^H]-thymidine ([Methyl-^3^H], Perkin Elmer, Waltham, MA)/well. NK cells were harvested onto Millipore filters, immersed in the scintillation fluid and the radioactive thymidine incorporation into NK-cell nucleus DNA was measured using an LKB Beta plate counter (Pharmacia, Gaithersburg, MD).

### Expansion of A-NK cells

Following culture in the presence of IL-2, A-NK cells were harvested, and their number and viability determined per mL of cell culture using Trypan blue dye exclusion assays and differential cell counting under an Olympus optical microscope. The expansion of A-NK cells was calculated by dividing the total numbers of A-NK cells at the end of their restimulation/culture with the initial A-NK-cell numbers determined after 5-h priming/adherence selection.

### Cytotoxicity assays

Standard 4-h ^51^Cr release cytotoxicity assays were performed in triplicates and four different effector to target (E:T) ratios against K562 cells as targets to measure NK activity, or Daudi cells as targets to measure LAK activity [10]. The data were presented ether as means of % Lysis of triplicates ± standard errors (SE), or as lytic units (LU)_20_ per 10^7^ effector cells and the total lytic units (total LU) per NK-cell culture. LU_20_ per 10^7^ was determined using the formula 107/(T x X20), where “T” is the number of target cells and “X20” is the estimated E:T ratio in which 20% of the target cells were killed. Total LU was calculated using the formula [(LU_20_ per 10^7^) x NK-cell number per culture]/10^7^.

### ELISA

The soluble IL-2Rα (CD25) levels were measured in the culture-conditioned media of A-NK and NA-NK cells using the enzyme-linked immunosorbent assay (ELISA) kit from R&D Systems, per manufacturer’s recommendations.

### Blocking IL-2 receptors after culture of A-NK cells

A-NK cells stimulated with 22 nM IL-2 for 10 or 14 days were harvested, washed, and re-cultured for 4 days in the presence 22 nM IL-2, and 20 μg/mL of isotype control IgG, anti-IL-2Rα and/or anti-IL-2Rβ monoclonal antibodies (mAbs). Following treatments, cells were assessed for proliferation using 4-h [^3^H]-thymidine incorporation assays.

### Blocking IL-2 receptors after priming of A-NK cells

A-NK cells primed with 22 nM IL-2 were cultured for 4 to 5 days in the presence of 0.022, 0.1 or 22 nM IL-2 ± 20 μg/mL of isotype control IgG, anti-IL-2Rα and/or anti-IL-2Rβ mAbs. After treatments, cells were assessed for proliferation, and for cytotoxicity, using 4-h ^51^Cr release assays against K562 and Daudi target cells.

### Apoptosis assay

A-NK cells were cultured in the presence of 22 nM IL-2 for 14 days, and incubated without or with 1 μg/mL of anti-Fas (APO-1) agonistic mAb and 2 μCi of [^3^H]-thymidine for 4 h. Afterward, the cell radioactivity was measured as described above for proliferation of A-NK cells.

### Statistical analysis

Means ± SE were computed where applicable. Statistical significances of data differences were calculated using a two-tailed Student’s *T-*test or ANOVA test. Differences were considered significant when the *P*-value was <0.05.

## Results

### A-NK cells cultured in nanomolar IL-2 concentrations exhibit functional impairment

**Figure 1.**
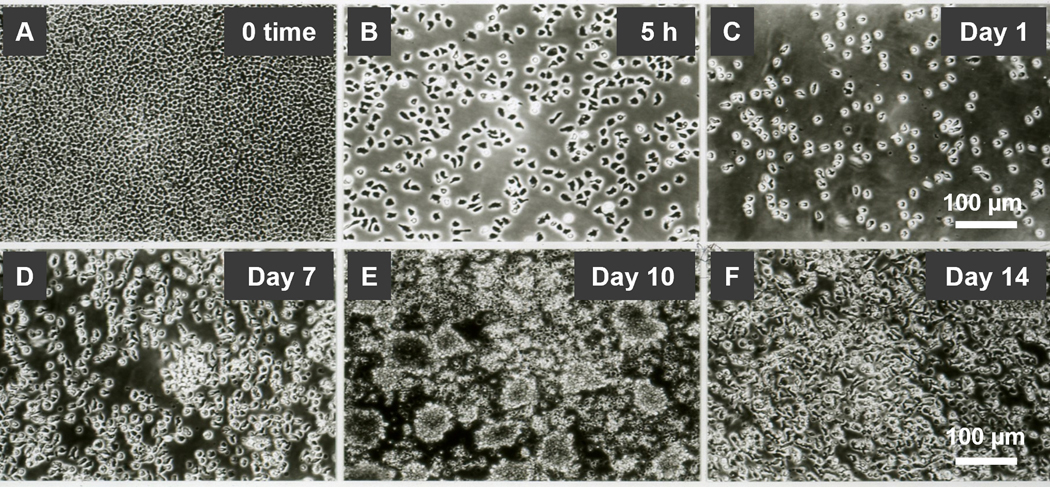
Generation and expansion of A-NK cells in the presence of 22 nM IL-2. (**A, B**) *Priming and adherence selection:* R-NK cells were suspended in CM supplemented with 22 nM IL-2 and incubated at 37°C for 5 h. (**A**) NK cells settle, forming a dense monolayer. (**B**) A fraction of these cells adheres to the culture plate surface. The adherent cells, characterized by various morphologies and membrane protrusions, represent primed A-NK cells. (**C-F**) *Restimulation and culture of primed A-NK cells:* Primed A-NK cells were supplied with fresh CM supplemented with 22 nM IL-2 and further cultured. (**C**) *Detachment of A-NK cells:* After 24 h of culture, A-NK cells detach and form a free-floating single-cell suspension. (**D**) *Initiation of active phase:* While remaining in suspension, A-NK cells begin to proliferate, forming a few small cellular clumps by day 7 of culture. (**E**) *Intensive active phase:* By day 10, the number of A-NK cells increases rapidly, forming numerous large cellular clumps. (**F**) *Functional decline:* By day 14, A-NK cells cease expansion and revert to a suspension of single, non-proliferating cells. Microphotographs were captured using an Olympus inverted microscope at 200x magnification. Scale bars represent 100 μm.

**Figure 2.**
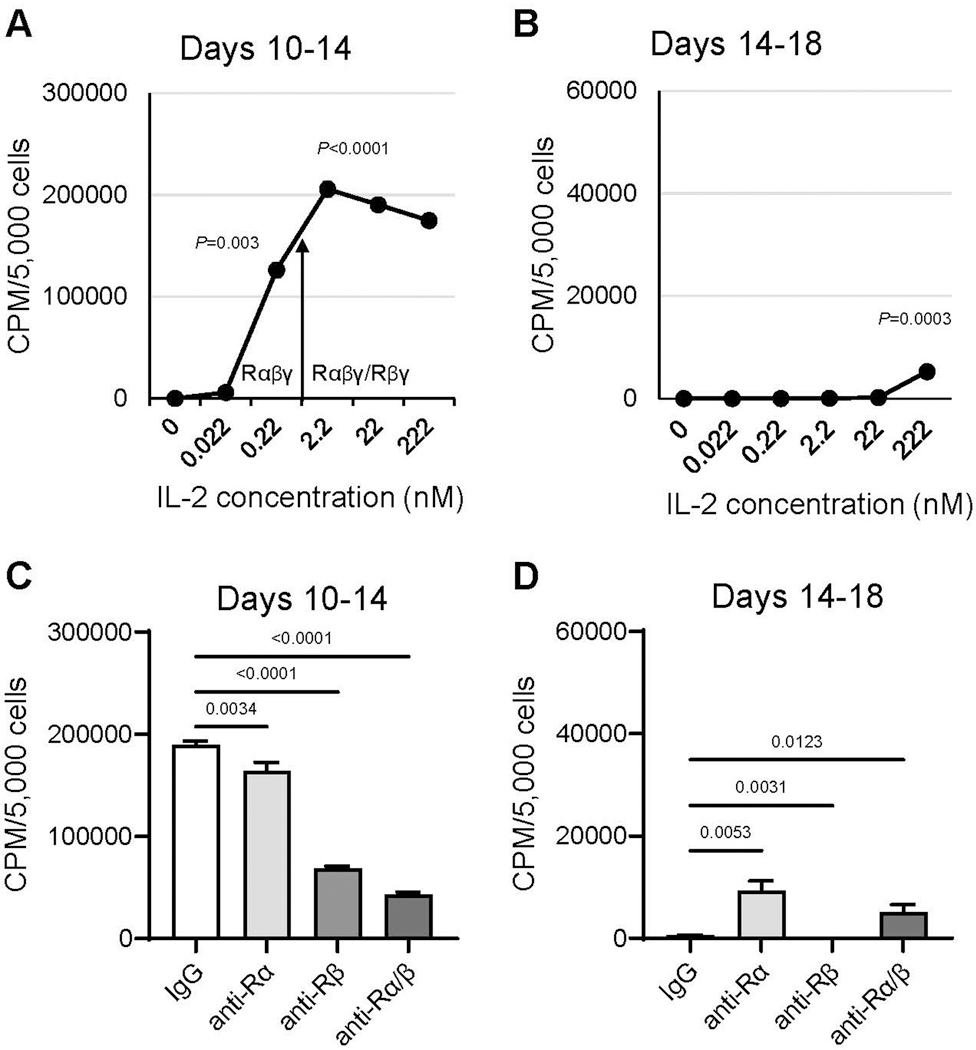
A-NK cell proliferation declines in the extended culture with nM IL-2. A-NK cells cultured in the presence of 22 nM IL-2 for either 10 or 14 days were subsequently re-cultured for additional 4 days with varying concentrations of IL-2 (**A, B**), or with 22 nM IL-2 and 20 μg/mL of isotype control IgG, anti-IL-2Rα and/or anti-IL-2Rβ mAbs (**C, D**). Following re-culture, A-NK cells were tested for proliferation using 4-h [^3^H]-thymidine incorporation assays. Data are presented as means CPM ± SE from four replicates. The arrow denotes the threshold of the IL-2 concentrations that selectively engage IL-2Rαβγ or both IL-2Rαβγ and IL-2Rβγ complexes. *P*-values represent the statistical significance of differences in A-NK cell proliferation in the absence or presence of IL-2 at the indicated concentrations (**A, B**), and in the presence of isotype control or anti-IL-2Rα and/or anti-IL-2Rβ mAbs (**C, D**).

A-NK cells undergo a series of sequential changes using the standard culture protocol. (i) First, CD56^low^CD16^low/−^ CD57^high^ANK-1^high^CD2^high^Integrins^high^ NK3 cells are primed and selected through adherence during 5-h incubation with 22 nM IL-2 [10,11] (Table 2, Figures 1A and 1B). (ii) This is followed by cell detachment and transition to a free-floating single-cell suspension after 24-h restimulation with 22 nM IL-2 (Figure 1C). (iii) Between days 7 and 10, the cells increasingly form dense clumps, accompanied by rapid expansion (Figures 1D and 1E). (iv) By day 14, the clumps dissociate into a single-cell suspension, and cell proliferation ceases (Figure 1F). Therefore, A-NK cells cultured using the standard protocol exhibit robust activity and expansion between days 7 and 10 of culture. However, their functions markedly decline by day 14. Importantly, A-NK cells cultured for 10 or 14 days in 22 nM IL-2 and subsequently re-cultured in pM (0.22 nM) or lower nM (2.2 nM) IL-2 concentrations for an additional 4 days retained high proliferative ability between days 10 and 14 of culture (Figure 2A). However, A-NK cell proliferation markedly declined between days 14 and 18 of culture, even in the presence of 222 nM IL-2 (Figure 2B). The high proliferation rate of A-NK cells from days 10 to 14 of culture was significantly inhibited by anti-IL-2Rα mAb (13.4%, *P*=0.0034), anti-IL-2Rβ mAb (68%, *P*<0.0001), or both mAbs (79%; *P*<0.0001) (Figure 2C). In sharp contrast, the poor proliferation of A-NK cells in 14- to 18-day culture was significantly increased (18-fold) by anti-IL-2Rα mAb, inhibited by anti-IL-2Rβ mAb (99.6%), and augmented by both these mAbs (8-fold), though to a lesser extent than with anti-IL-2Rα mAb alone (Figure 2D). These results indicate that A-NK cells cultured for 10 days in nM IL-2 concentrations can grow quite well in pM IL-2 concentrations. In contrast, the growth of A-NK cells continuously cultured in 22 nM IL-2 for 14 days declined, even after their transfer to medium supplemented with 222 nM IL-2. These data further suggest that IL-2 receptor expression or function are altered by day 14 of culture. Additionally, A-NK cells cultured for 14 days expressed CD57 (a marker of both NK3 and terminally differentiated NK cells [6,8, 13,14]), whereas A-NK cells cultured for 10 days, and NA-NK cells cultured for both 10 and 14 days did not (Table 3). Furthermore, A-NK cells cultured for 14 days were highly susceptible to both IL-2 withdrawal and Fas-mediated apoptosis (data not shown). Taken together, these data indicate that A-NK cells cultured for 14 days in the nM IL-2 concentrations become unresponsive to IL-2, terminally differentiated, dysfunctional and likely therapeutically incompetent.

### A-NK cells generated in nM IL-2 exhibit prolonged expression of IL-2Rα

**Figure 3.**
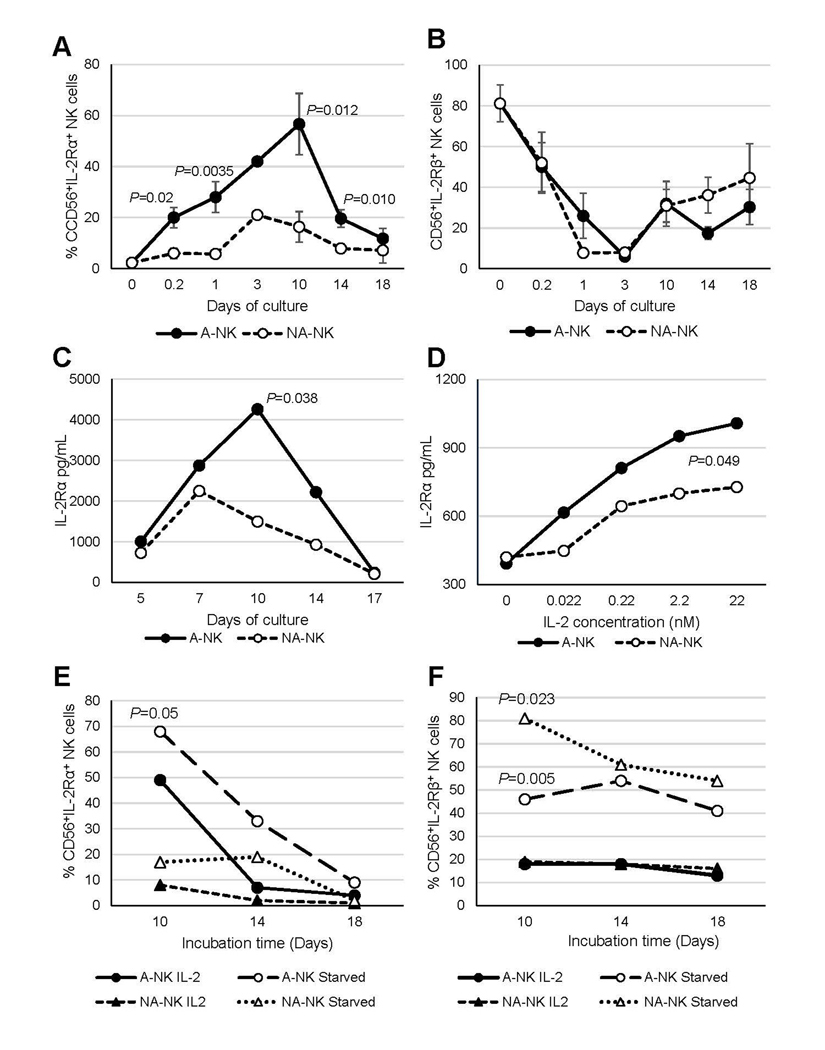
Expression and shedding of IL-2Rs by A-NK and NA-NK cells. (**A, B**) *IL-2R expression:* A-NK and NA-NK cells collected at various time point during priming and culture were analyzed, along with R-NK cells for surface expression of IL-2Rα (**A**) and IL-2Rβ (**B**) using flow cytometry. Data are presented as mean percentages ± SE of receptor positive cells from three to ten independent experiments. *P* values denote the statistical significance of differences in IL-2Rα expression between A-NK cells and NA-NK cells at the indicated time points. (**C, D**) *Shedding of IL-2Rα:* (**C**) Primed A-NK cells and NA-NK cells were cultured with 22 nM IL-2 for varying durations, followed by re-culturing in fresh CM supplemented with 22 nM IL-2 for additional 2 days. (**D**) Primed A-NK cells and NA-NK cells were cultured with varying IL-2 concentrations for 5 days. Cell-conditioned media from these cultures were then collected and analyzed for soluble IL-2Rα levels using ELISA. Data shown are representative of two independent experiments. Results are expressed as mean concentrations (pg/mL) from triplicate samples. SEs were <5% of the means. *P* values indicate the statistical significance of differences in soluble IL-2Rα levels between A-NK-cell and NA-NK-cell cultures. (**E, F**) *Receptor expression following IL-2 withdrawal:* A-NK and NA-NK cells were cultured for 10, 14 and 18 days in the presence of 22 nM IL-2, then re-cultured for 4 h either with or without IL-2 (22 nM). After this cytokine withdrawal period, surface expression of IL-2Rα and IL-2Rβ was assessed by flow cytometry. The data are percentages of A-NK cells and NA-NK cells expressing IL-2Rα and IL-2Rβ. *P* values indicate the statistical significances of differences between A-NK cells and NA-NK cells incubated with or without IL-2.

The previous finding that nM IL-2-primed NK3 (A-NK) cells express the IL-2Rα mRNA (12), along with our current observation that 10-day A-NK cells cultured in nM IL-2 concentrations resume efficient proliferation upon transfer to media containing pM IL-2 (Figure 2A), suggests that A-NK cells acquire expression of the high-affinity IL-2Rαβγ complex. In contrast, the markedly impaired proliferation of A-NK cells after 14 days of culture, even in high nM IL-2 concentrations (Figure 2B), indicates that prolonged exposure to nM IL-2 may lead to downregulation of IL-2 receptor expression. We tested these possibilities by investigating the cell-surface expression and shedding of IL-2 receptors by A-NK and NA-NK cells at different time points of their generation, from 5 hours to 18 days (Figure 3). We found that approximately 20% of A-NK cells exhibited *de novo* expression of IL-2Rα during a 5-h priming with 22 nM IL-2. Subsequent culture in 22 nM IL-2 led to a gradual increase in the frequency of IL-2Rα^+^ A-NK cells, reaching 28%, 42% and a maximum of 57% on days 1, 3 and 10 of culture, respectively. The increase in the frequency of IL-2Rα^+^A-NK cells correlated with increased activity of these cells. By days 14–18, when A-NK cells become unresponsive to IL-2, the frequency of IL-2Rα^+^A-NK cells declined to 12% (Figure 3A). In contrast to A-NK cells, only 6% of NA-NK cells were IL-2Rα^+^ during the 5-h priming with 22 nM IL-2, and low frequency persisted throughout the 18-day culture (Figure 3A). Thus, the frequency of IL-2Rα^+^A-NK cells remained consistently and significantly higher than that of IL-2Rα^+^ NA-NK cells at all time points during culture. In contrast, the frequency of IL-2Rβ^+^ A-NK and NA-NK cells similarly and markedly declined from 80% at time 0 to 10% by day 3 of culture, but recovered to 45% by day 18 of culture (Figure 3B).

A-NK cells also exhibited elevated IL-2Rα shedding from the cell surface, as indicated by increased levels of soluble IL-2Rα in culture supernatants (Figures 3C and 3D). Shedding of IL-2Rα correlated with the changes in the frequency of IL-2Rα^+^A-NK cells and their activity, peaking on day 10 of culture and then rapidly declining by days 14 and 17 (Figure 3C). The rate of receptor shedding was dependent on the IL-2 concentration, being threefold higher in 22 nM compared to 0.022 nM IL-2 (Figure 3D). Furthermore, both the magnitude and kinetics of IL-2Rα shedding differed significantly between A-NK and NA-NK cell cultures. Across various time points and IL-2 concentrations, A-NK cells consistently showed higher levels of IL-2Rα shedding than NA-NK cells (Figures 3C and 3D).

The expression of IL-2 receptors on the cell surface may be downregulated through IL-2-induced enzymatic shedding and internalization [24,25]. To minimize these processes and accurately detect IL-2R^+^ A-NK and NA-NK cells, we examined these cells following re-culture in IL-2-free CM [i.e., cytokine withdrawal (Figures 3E and 3F)]. Cytokine starvation significantly enhanced the frequency of IL-2Rα^+^ A-NK and NA-NK cells (e.g., in day-10 cultures, from 49% to 70%, and from 8% to 17%, respectively), as well as IL-2Rβ^+^ cells (e.g., in day-10 cultures, from 18% to 46%, and from 19% to 81%, respectively). Notably, IL-2 withdrawal more prominently enhanced IL-2Rα expression in A-NK cells (Figure 3E), whereas it more substantially increased IL-2Rβ in NA-NK cells (Figure 3F). These results demonstrate that A-NK cells primed with nM IL-2 rapidly and persistently express IL-2Rα *de novo*, alongside constitutive IL-2Rβ expression, and that IL-2Rα levels in cultures maintained with nM IL-2 correlate with A-NK cell function.

### A-NK cells primed with nM IL-2 function optimally in pM IL-2

**Figure 4.**
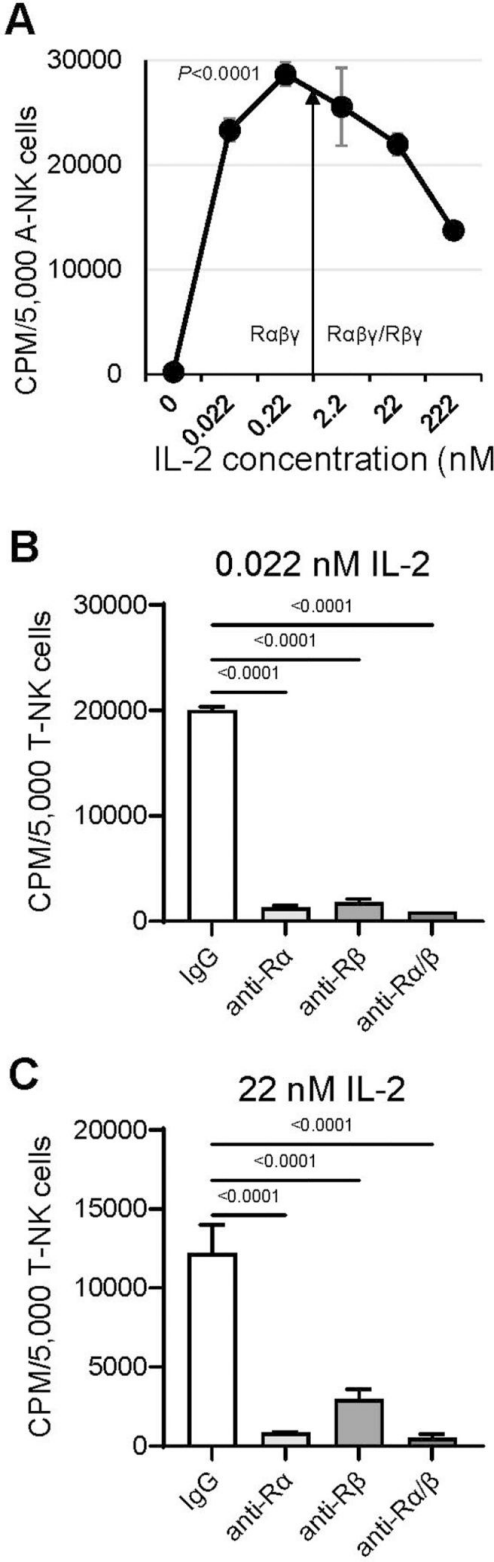
A-NK cells proliferate efficiently in pM IL-2 (A) and this activity is inhibited by anti-IL-2Rα mAb (B, C). (**A**) A-NK cells were primed with 22 nM IL-2, then cultured with various IL-2 concentrations for 5 days. Upon this restimulation, proliferation of A-NK cells was assessed using a 4-h [^3^H]-thymidine incorporation assay. The arrow indicates the threshold of the IL-2 concentrations that selectively engage IL-2Rαβγ, or both IL-2Rαβγ and IL-2Rβγ complexes. Representative data from one of four similar experiments are shown. Data are expressed as the mean CPM ± SE from six replicates. *P* value indicates statistical significance of differences between A-NK cells cultured in 0.22 nM (222 pM) IL-2 and those cultured in 22 or 222 nM IL-2. (**B, C**) Primed A-NK cells were cultured for 5 days with either pM or nM IL-2 in the presence of 20 μg/mL of an isotype control IgG, anti-IL-2Rα, anti-IL-2Rβ or a combination of the two mAbs. Following this culture, A-NK cell proliferation was tested using [^3^H]-thymidine incorporation assays. The data presented are representative of two similar experiments. Results are expressed as the mean CPM ± SE from six replicates.

**Figure 5.**
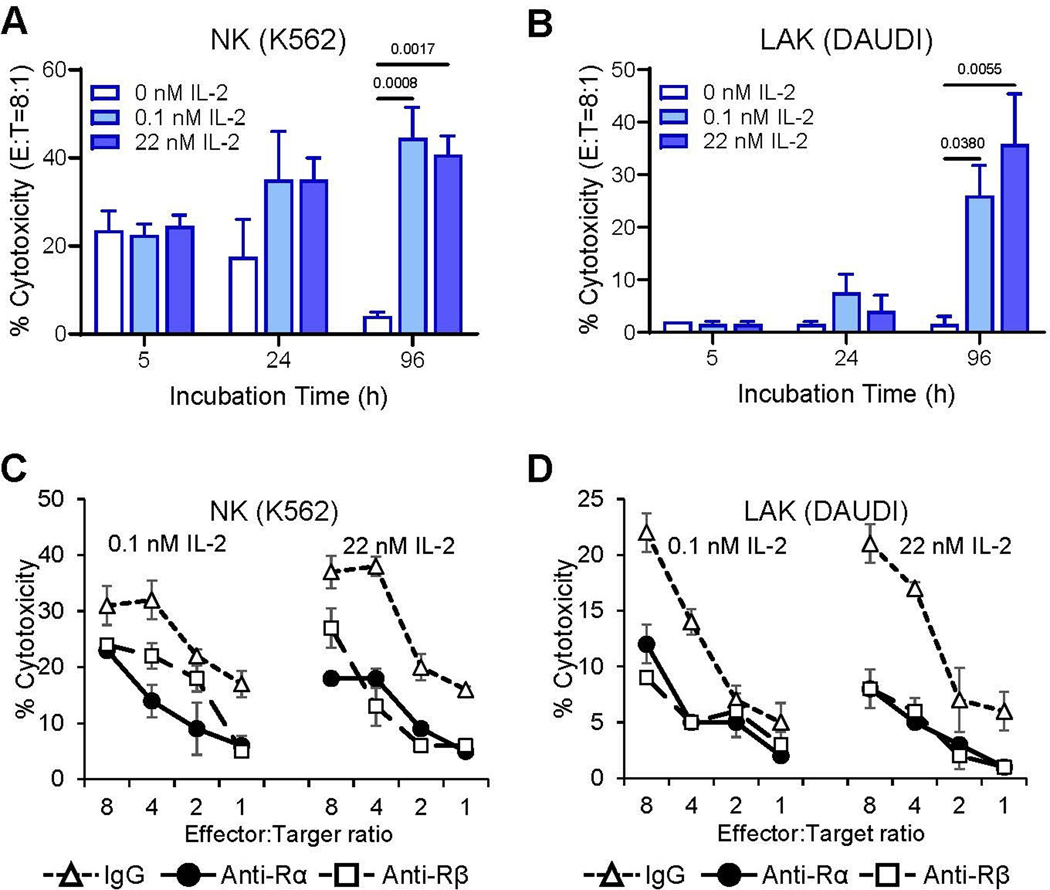
A-NK cells cultured in pM IL-2 enhance NK cytotoxicity and develop robust LAK activity (A, B), both of which are inhibited by anti-IL-2Rα mAb (C, D). Following priming, A-NK cells were cultured either with 0.11 or 22 nM IL-2 for 5 h, 24 h or 96 h (**A, B**), and/or with 20 μg/mL of isotype control IgG, anti-IL-2Rα or anti-IL-2Rβ mAbs for 96 h (**C, D**). After the indicated culture periods, A-NK cells were assessed for NK cytotoxicity against K562 and LAK activity against DAUDI target cells using 4-h ^51^Cr release assays. (**A, B**) Data represent means % cytotoxicity ± SE from two to four independent experiments. (**C, D**) Data are means % cytotoxicity ± SE from triplicate samples in one representative experiment of two performed.

We next investigated whether *de novo* expression of IL-2Rα on nM IL-2-primed A-NK cells leads to formation of the functional high-affinity IL-2Rαβγ complex, therefore enabling these cells to function effectively in pM IL-2 concentrations. To address this, we assessed the proliferation (Figure 4), NK cytotoxicity and LAK activity (Figure 5) of A-NK cells that were primed with 22 nM IL-2 and subsequently restimulated/cultured in either pM or nM IL-2 concentrations for 4 to 5 days, without or with isotype control IgG or blocking mAbs targeting IL-2Rα and IL-2Rβ. A-NK cells proliferated vigorously in both pM and nM IL-2. Notably, they developed robust proliferative activity in 0.022 nM (22 pM) and, especially, in 0.22 nM (222 pM) IL-2, displaying better proliferation in pM than in nM IL-2 (Figure 4A). Co-culture with anti-IL-2Rα and/or anti-IL-2Rβ blocking mAbs resulted in near-complete inhibition of A-NK cell proliferation over five days in both pM and nM IL-2 concentrations (Figure 4B).

Consistent with their robust proliferative responses to both pM and nM IL-2, A-NK cells primed with nM IL-2 exhibited enhanced NK cytotoxicity and developed potent LAK activity in cultures containing either pM or nM IL-2 (Figures 5A and 5B). Furthermore, treatments with either anti-IL-2Rα or anti-IL-2Rβ mAbs, compared to isotype control IgG, significantly inhibited both the enhancement of NK activity (Figure 5C) and the development of LAK activity (Figure 5D) in A-NK cells restimulated with either pM or nM IL-2. These results suggest that *de novo* expression of IL-2Rα on nM IL-2-primed A-NK cells facilitates the assembly of the functional high-affinity IL-2Rαβγ complex through its association with the constitutively expressed β and γ receptor subunits. This newly assembled receptor complex binds pM concentrations of IL-2 and effectively transduces signals that drive robust proliferation and anti-tumor activity in A-NK cells.

### A-NK cells primed with nM IL-2 maintain expression of IL-2Rα and anti-tumor activities in cultures with pM IL-2

We next evaluated the persistence of A-NK cell proliferation and anti-tumor activity in prolonged cultures supplemented with pM concentrations of IL-2. Notably, after 14 days in 0.22 nM (222 pM) IL-2, 50% of A-NK cells expressed IL-2Rα, opposing only 6% to 14% of cells cultured in 22 or 222 nM IL-2. In contrast, only 4% to 8% of NA-NK cells exhibited IL-2Rα expression under either culture condition (Supplementary Figure 1A). Conversely, 68% of both A-NK cells and NA-NK cells cultured in 0.22 nM IL-2 expressed IL-2Rβ, while only 10% did so in 22 and 222 nM IL-2 (Supplementary Figure 1B). These data suggest that long-term culture in pM IL-2 supports high-affinity IL-2R expression in and functional competence of A-NK cells, whereas standard nM IL-2 culture leads to receptor downregulation and loss of function.

To further examine this, A-NK cells primed with 22 nM IL-2 were either continuously cultured in 22 nM IL-2 (standard protocol) or cultured in 0.022 nM (22 pM) IL-2 for 2, 4, 5, 6, 8 or 10 days before re-culture with 22 nM IL-2. All A-NK cell groups were cultured for a total of 13 days before assessments. Regardless of regimen, A-NK cells consistently co-expressed CD56 and CD16 (Table 4). Importantly, on the culture-day 13, IL-2-mediated functions, including proliferation, enhanced NK cytotoxicity and LAK activities, were robust and comparable across all groups of A-NK cells that had been exposed to pM IL-2 for varying durations (Table 4). These findings demonstrate that A-NK cells primed in nM IL-2 can retain high-affinity IL-2R expression and IL-2-dependent functions during extended culture in pM IL-2.

## Discussion

NK cells briefly stimulated with nM IL-2 have demonstrated considerable potential for cancer treatment in both experimental murine models and clinical trials [18,26–30]. In contrast NK cells subjected to prolonged activation with nM IL-2 have shown limited efficacy in clinical settings ([31], our unpublished findings). The present study shows that A-NK cells generated under sustained, high-concentration IL-2 stimulation undergo terminal differentiation, functional impairment, and have reduced survival capacity, ultimately rendering them incapable of mediating effective antitumor responses. Extended stimulation with nM IL-2, commonly used in the generation of therapeutic NK cells [32,33], represents a non-physiological condition that may induce NK cell disfunction.

We investigated an alternative strategy that more closely resembles physiological conditions and has the potential to generate functionally and therapeutically competent NK cells. Building on our previous observation that NK3 cells, upon priming/adherence selection in nM IL-2, *de novo* express the IL-2Rα transcript [12], we now demonstrate that these A-NK cells also *de novo* express IL-2Rα protein, thereby assembling the high affinity IL-2αβγ receptor complex. As a result, upon restimulation with pM IL-2, they vigorously proliferate and generate functionally competent anti-tumor effector cells. Moreover, when subsequently cultured in pM IL-2 concentrations, A-NK cells maintain high levels of IL-2Rα expression, robust and sustained proliferation, as well as enhanced NK cytotoxicity and potent LAK activity over extended periods. Our results demonstrate that A-NK cells generated using this newly established protocol, which includes priming with nM IL-2 followed by restimulation/culture in pM IL-2, are long-lived, functionally competent, exhibit potent anti-tumor activity, and display reduced IL-2 dependency. These attributes make them promising candidates for therapeutic applications.

Sustained culture of A-NK cells in the presence of high IL-2 concentrations persistently engages both the high- and the intermediate-affinity IL-2 receptors, resulting in chronic hyperactivation. This hyperactivation may trigger strong feedback mechanisms that alter A-NK-cell function. A major consequence of such chronic stimulation is the progressive loss of A-NK cell responsiveness to IL-2. One key mechanism underlying this dysfunction appears to be the downregulation of cell-surface IL-2 receptor expression, particularly IL-2Rα, leading to disruption of the high-affinity IL-2Rαβγ complex. This downregulation is likely driven by extensive shedding of the receptor ectodomain, mediated by increased activity of the metalloproteinases ADAM10 and ADAM17 [25], which may be induced by nM concentrations of IL-2 (Figure 3D).

An alternative, though not mutually exclusive, explanation is that IL-2 unresponsiveness in A-NK cells is mediated by the cytokine-induced suppressors of cytokine signaling (SOCS) family proteins, which are potent negative regulators of JAK/STAT-mediated cytokine signaling [34]. This hypothesis is supported by our observation that A-NK cells, after prolonged hyperactivation and loss of IL-2 responsiveness, respond to non-cytokine stimuli induced by the anti-IL-2Rα antibody. This suggests the potential activation of alternative signaling pathways via crosslinking of IL-2Rαβγ and/or engagement of Fc receptors, thereby circumventing the inhibited canonical cytokine signaling cascade. While this is an appealing mechanism for A-NK cell dysfunction following chronic exposure to high IL-2 concentrations, it remains speculative in the absence of direct evidence at the protein or signaling level. Future studies incorporating phospho-STAT analysis and SOCS inhibition will be necessary to rigorously test this hypothesis. Furthermore, considering that nM IL-2 elicits substantial release of soluble IL-2Rα by A-NK cells, it is plausible that this soluble receptor also contributes to IL-2 unresponsiveness by sequestering IL-2 and preventing its interaction with membrane-bound IL-2 receptors.

While approximately 80% of IL-2-primed NK3 (A-NK) cells express IL-2Rα mRNA [12], only about 20% initially express the corresponding receptor protein. However, the fraction of A-NK cells expressing the receptor protein gradually increases during continued culture with IL-2, reaching levels comparable to mRNA expression by day 10. Notably, the pM IL-2-induced proliferation of A-NK cells correlates with the proportion of cells expressing IL-2Rα protein. Thus, proliferation in pM IL-2 is substantial from 5 hours to 5 days of culture (Figure 4A), but increases several-fold between days 10 and 14 (Figure 2A). The observed lack of correlation between IL-2Rα mRNA expression and IL-2Rα protein levels in A-NK cells at the time of priming may suggest either rapid turnover of the receptor protein, or, more likely, heterogeneity within the A-NK cell population with respect to IL-2Rα expression and their capacity to proliferate in response to pM IL-2. The latter interpretation is supported by our findings that a similar and relatively low proportion (20–30%) of early A-NK cells express the IL-2Rα protein (present study) and incorporate [^3^H]-thymidine [10].

Both the proliferation and tumoricidal activity of nM IL-2-primed A-NK cells can be similarly enhanced by restimulation with either pM or nM IL-2, and similarly inhibited with anti-IL-2Rα and anti-IL-2Rβ blocking antibodies. However, while proliferation is almost completely inhibited (94–96%), cytotoxicity is only partially reduced (37–71%). These findings further support the notion that A-NK cells are heterogeneous, consisting of two functionally distinct subtypes. The first subtype rapidly and persistently expresses IL-2Rα *de novo*, forms the high-affinity IL-2Rαβγ complex upon nM IL-2 priming, and subsequently undergoes robust proliferation and amplification of tumoricidal activity in response to pM IL-2. In contrast, the second subtype lacks IL-2Rα expression and responds selectively to nM IL-2 by enhancing tumoricidal activity without proliferating. These putative subsets may represent distinct differentiation stages within the NK3 lineage. Future comprehensive studies utilizing high-dimensional single-cell profiling, including single-cell RNA sequencing and mass cytometry [8], will be conducted to further investigate this hypothesis and determine whether the observed functional subsets indeed reflect progressive differentiation within the NK3 lineage or represent stable, functionally distinct populations.

Because A-NK cells are derived from NK3 (adaptive) cells, are generated through cytokine priming, acquire *de novo* and sustained expression of IL-2Rα, and maintain long-term functional competence in pM concentrations of IL-2, enabling them to mediate antitumor responses [16,18–20], they closely resemble memory-like NK cells [9,35–39]. Therefore, it is plausible to speculate that A-NK cells represent an enriched population of memory-like NK cells, with NK3 cells and their early progeny (i.e., nM IL-2-primed A-NK cells) serving as their precursors.

The finding that priming NK3 cells with nM IL-2 induces their differentiation into A-NK cells, which remain functionally competent for extended periods when cultured with pM IL-2, has critical implications for adoptive NK cell therapy. Collectively, our data introduce a novel and superior method of generating durable and therapeutically potent A-NK cells. Because nM IL-2-primed A-NK cells endogenously secrete IL-2 and may sustain their activity through autocrine and paracrine mechanisms [12], they may not only survive and function efficiently over prolonged time in the presence of low (pM) levels of exogenous IL-2 but may also persist in its absence. Consequently, A-NK cells generated via this approach could maintain viability and effector function *in vivo* for prolonged periods, even with minimal or no exogenous IL-2 support.

The use of optimized A-NK cells in adoptive immunotherapy offers a highly promising strategy for cancer treatment. These cells demonstrate potent and sustained proliferative and tumoricidal activities, while requiring only minimal concentrations of exogenous IL-2. This markedly reduced IL-2 dependency presents a significant therapeutic advantage by mitigating the dose-limiting toxicities typically associated with high-dose IL-2 regimens [34,40–43]. By harnessing the intrinsic functional resilience under low IL-2 conditions, this approach has the potential to substantially improve both the safety and efficacy of NK cell-based immunotherapies in clinical practice. To evaluate these translational prospects, ongoing *in vivo* preclinical studies in our laboratory are assessing the persistence, trafficking, and anti-tumor activity of A-NK cells expanded under optimized picomolar IL-2 conditions. Concurrently, we are advancing protocols for the GMP-compliant large-scale manufacturing of optimized A-NK cells, including those derived from cancer patients, using serum-free reagents and closed-system culture platforms. Importantly, the minimal IL-2 requirement not only improves the therapeutic applicability of this strategy but also markedly reduces production costs, further distinguishing this strategy from conventional NK cell manufacturing approaches.

Our findings also suggest that optimal *in vivo* generation, expansion, and sustained maintenance of functionally competent activated endogenous NK3 (A-NK) cells, capable of mediating effective and low-toxicity cancer therapy, may be more efficiently achieved through a brief high-dose IL-2 pretreatment [to prime NK3 (A-NK) cells], followed by an extended low-dose IL-2 treatment (to support A-NK cell expansion and function), rather than by conventional multi-day bolus or continuous high-dose IL-2 infusions [34,40–43]. We are also currently testing this possibility in animal models.

## Supplementary Material

JCAI-25-110-Supplementary-File

## Figures and Tables

**Table 1.** Characteristics of NK cell subpopulations in human peripheral blood.^a^

Markers	NK1 cluster	NK2 cluster	NK3 cluster
***Adhesion molecules***			
C56 (140-kDa N-CAM)	Low	High	Low
ANK-1 (180-kDa N-CAM)	Absent	Absent	High
CD57	Low	Absent	High
CD2	Low	Absent	High
Integrins	Low	Low	High
Vimentin	Low	Low	High
***Ectoenzyme***			
CD38	High	Low	Low
***Receptors***			
CD16	High	Absent	Low/Absent
CD158b	Low	Low	High
CD158e1	Low	Low	High
CD161	High	Low	Low
CD94/NKG2A	Absent	High	Absent
NKG2D	Low	High	Low/Absent
NKP46	Absent	High	Low
CD49d	Low	Low	High
CX3CR1	High	Low	Low/Absent
CCR7	High	Low	Low
CCR5	Low	Low	High
CD117 (c-kit)	Absent	High	Absent
CD27	Low	High	Low
CD271 (NGFR)	Absent	Low	High
IL-7R	Low	High	Low
IL-2Rγ	High	High	High
IL-2Rβ	High	High	High
IL2-Rα	Absent	High	Absent
***Cytotoxic molecules***			
Perforin	High	Absent/Low	High
Granzyme B	High	Absent/Low	High
***Gene Expression***			
*CD3E*	Absent	Absent	High
*NKG7*	High	Low	Absent
*GZMH*	Absent	Absent	High
*PRDM1*	Low	Low	High
*IL32*	Low	Low	High
*CCL4*	High	Low	Low
*CCL5*	Low	Low	High
***Functions***			
NK Cytotoxicity	High	Low	High
ADCC	High	Low/Absent	Low
Induced LAK activity	High	Low	High
Induced proliferation	Low	High	High
Induced cytokine production	Low	High	High
Response to nM IL-2	High	High	High
Response to pM IL-2	Absent	High	Absent

a Lanier L, 2024[1]; JacquelotN, *et al.* 2022 [2]; Freud AG, *et al.* 2017 [6]; Laskowski TJ, *et al.* 2022 [7]; Rebuffet L, *et al.* 2024 [8]; Vujanovic NL, *et al.* 1993 [10]; Vitolo D, *et al.* 1993 [11]; Li S, *et al.* 2004 [12].

**Table 2.** A-NK cells during priming/adherence display a basic NK3 phenotype.^a^

*Integrins*	Marker	Measure	No. Exp.	R-NK	A-NK	NA-NK
	CD56	MFI	3	120±37	70±14*	102±27
	CD16	MFI	4	241±48	145±10*	205±29
	CD57	MFI	2	410±51	451±46	386±47
	ANK-1	MFI	3	516±62	657±170	426±52
	CD57^+^	Freq. (%)	3	55±4	87±7*	50±5
	ANK1^+^	Freq. (%)	3	55±11	85±4*	49±12
	CD2^+^	Freq. (%)	1	73	92	70
	*CD29*	MFI	1		83	56
	*CD49d*	MFI	1		171	86
	*CD18*	MFI	2		273±0.7	216±2.8
	*CD11a*	MFI	2		344±0.7	285±7.1
	*CD11b*	MFI	2		531±0.3	367±8.5

a Resting NK (R-NK) cells were treated with the protein synthesis inhibitor cycloheximide (10 μg/mL) for 1 h, to preserve their original phenotype during priming. Following this treatment, R-NK cells were induced with 22 nM IL-2 for 1 h. Adherent NK (A-NK) and non-adherent NK (NA-NK) cells were harvested separately. R-NK, A-NK and NA-NK cells were stained with fluorochrome-conjugated mAbs and analyzed for expression of the listed markers using flow cytometry.

* Statistically significant differences between A-NK and R-NK or NA-NK cells (*P*=0.012–0.05).

**Table 3.** CD57 is expressed by 14-day, but not 10-day-cultured A-NK cells.^a^

Marker	A-NK		NA-NK	
	10-day	14-day	10-day	14-day
% CD56^+^	90	90	85	92
% CD57^+^	12	93	7	1

a A-NK and NA-NK cells were stained with fluorochrome-conjugated anti-CD3, anti-CD56 and anti-CD57 antibodies and analyzed by flow cytometry.

**Table 4.** A-NK cells cultured with pM IL-2 retain functionality in long-term cultures.^a^

		Culture day for adding 22 nM IL-2						
	Measure	0	2	4	5	6	8	10
^b^CD3^−^CD56^+^	Freq. (%)	92	91	95	93	94	91	88
^c^CD56^+^CD16^+^	Freq. (%)	86	90	91	84	80	78	54
^d^Proliferation	CPM	65,922	57,709	67,124	61,583	48,325	42,247	44,242
^e^K562	LU_20_/10^7^ NK	5,121	7,021	3,765	4,987	6,665	6,476	6,011
^f^DAUDI	LU_20_/10^7^ LAK	1,654	2,352	2,230	1,687	2,541	2,019	2,970

a A-NK cells primed for 5h with 22 nM IL-2 were restimulated/cultured in 0.022 nM IL-2. On days 0, 2, 4, 5, 6, 8, and 10 of this culture, cells were supplemented with 22 nM IL-2 and cultured until day 13, when they were tested.

b,c Flow cytometry data are from a single experiment.

d-f Proliferation and cytotoxicity data represent means from 2 experiments.

d SE=513–3,330

e SE=867–3,930

f SE=578–2,236

## Abbreviations:

ILCs: Innate Lymphoid Cells
NK: Natural Killer
IL: Interleukin
A-NK: Adherent NK
NA-NK: Non-Adherent NK
R: Receptor
pM: Picomolar
nM: Nanomolar
ADCC: Antibody-Dependent Cell-mediated Cytotoxicity
LAK: Lymphokine-Activated Killer
