# Supplementary material for: Adherent Natural Killer Cells De Novo Express IL-2Rα and Sustain Long-Lasting, Potent Anti-Tumor Activity in Picomolar Concentrations of IL-2
Source: J Cancer Immunol (Wilmington). Author manuscript; Available in PMC 2026 Mar 27. (PMC13021156; doi:10.33696/cancerimmunol.7.110)
Supplement: JCAI-25-110-Supplementary-File [file NIHMS2109967-supplement-JCAI-25-110-Supplementary-File.pdf]

Vujanovic NL, Vujanovic L, Whiteside TL. Adherent Natural Killer Cells De Novo Express IL-2Rα and Sustain Long-Lasting, Potent Anti-Tumor Activity in Picomolar Concentrations of IL-2. J Cancer Immunol. 2025;7(3):109–122.

Supplemental Table 1. Reagents and antibodies.

**Reagents**	**Company source**	**Address**
Recombinant human IL-2	Chiron	Emeryville, CA
CD25 (IL-2Rα) ELISA kit	R&D Systems	Minneapolis, MN
Cycloheximide	Sigma-Aldrich	St. Luis, MO
**Antibodies**	**Company source**	**Address**
Cy-chrome-conjugated anti-human CD3 mAb	BD Biosciences	San Jose, CA
Phycoerythrin (PE)-conjugated anti-human CD56, CD16, CD3, CD14, CD19, CD25 mAbs	BD Biosciences	San Jose, CA
Fluorescein isothiocyanate (FITC)-conjugated CD3, CD16, CD14, CD19, CD57, CD2, CD29, CD49d, CD18, CD11a, CD11b, CD11c, CD122 (IL-2Rβ) mAbs	BD Biosciences	San Jose, CA
FITC-conjugated anti- ANK-1 (8G6) mAb	Absolute Antibody	Boston, MA
Fluorochrome-conjugated isotype-control mAbs	BD Biosciences	San Jose, CA
Anti-human CD122 (IL-2Rβ, TU27) and CD25 (IL-2Rα, B-B10) blocking mAbs	Thermo-Fisher Scientific	Waltham, MA
Isotype-control mAbs	Thermo-Fisher Scientific	Waltham, MA
Agonistic anti-human Fas (CD95) APO-1 mAb	Sigma-Aldrich	St. Luis, MO

Vujanovic NL, Vujanovic L, Whiteside TL. Adherent Natural Killer Cells De Novo Express IL-2Rα and Sustain Long-Lasting, Potent Anti-Tumor Activity in Picomolar Concentrations of IL-2. J Cancer Immunol. 2025;7(3):109–122.

Suppl. Fig. 1

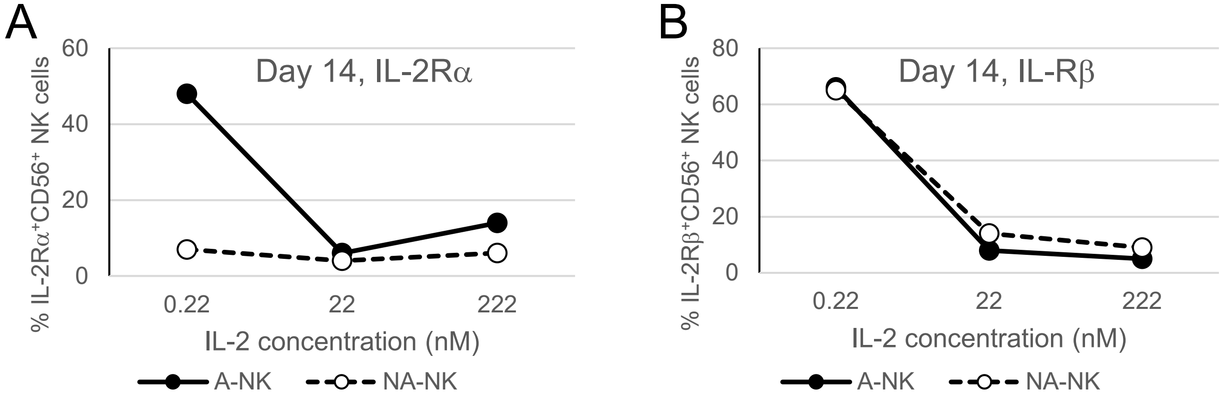

Supplemental Figure 1. *A-NK cells primed with nM IL-2 and restimulated with pM IL-2 maintain prolonged expression of both IL-2Rα and IL-2Rβ.* Primed A-NK cells and NA-NK cells were cultured in 0.22, 22 or 222 nM IL-2 for 14 days. At the end of the culture period, surface expression of IL-2Rα and IL-2Rβ was assessed by flow cytometry. Presented data are percentages of A-NK and NA-NK cells expressing each receptor.
